# Transfer strategy performance varies with embryo morphokinetic quality in good prognosis IVF/ICSI cycles

**DOI:** 10.3389/fendo.2026.1906497

**Published:** 2026-08-18

**Authors:** Mariabeatrice Dal Canto, Elena Bartoli, Marco Vitali, Monia Lain, Elena De Ponti, Claudio M. M. Brigante, Mario Mignini Renzini, Davide Perruzza, José Buratini

**Affiliations:** 1 Biogenesi, Reproductive Medicine Centre, Istituti Clinici Zucchi, Monza, Italy; 2 Department of Medical Physics, Fondazione IRCCS San Gerardo dei Tintori, Monza, Italy; 3 Department of Structural and Functional Biology, Institute of Biosciences, São Paulo State University, Botucatu, Brazil

**Keywords:** cleavage stage embryo transfer, embryo morphokinetics, freeze-all, live birth, miscarriage

## Abstract

Whether embryo morphokinetic quality influences the clinical efficacy of different embryo transfer (ET) strategies in good prognosis ICSI/IVF cycles remains an open question. This study evaluated the impact of early morphokinetic selection on clinical outcomes following fresh cleavage-stage single embryo transfers (FC-SETs) versus cryopreserved blastocyst single embryo transfers (CB-SETs). We conducted a retrospective cohort study analyzing 925 single embryo transfers, which included 565 FC-SETs and 360 CB-SETs. Embryos were ranked using an in-house Kinetic Evolution algorithm (KINEVO), whose performance was assessed through the relationship between KINEVO scores and live birth rates (LBR) following FC-SETs. Subsequently, clinical outcomes were compared across three distinct embryo cohorts: overall (n=925), top-quality (n=610) and non-top-quality (n=315). Pregnancy and live birth rates following FC-SETs were 57% and 51% higher (p ≤ 0.001) in A-score embryos as compared to non-A embryos. Multivariate analysis confirmed that the KINEVO score (OR = 1.62; p=0.015) and maternal age (OR = 0.88; p<0.0001) were independently associated with live birth. Overall, CB-SETs provided a higher clinical pregnancy rate (47.5% vs. 35.6%; p<0.0001) but a significantly higher miscarriage rate (21.6% vs. 9.5%; p=0.001) compared to FC-SETs. When a top-quality embryo was transferred, CB-SETs provided a numerically higher CPR (47.5% vs. 41.4%; p = 0.082) but a significantly higher miscarriage rate (21.4% vs. 10.5%; p = 0.011) as compared to FC-SETs, resulting in comparable live birth rates (37.4% vs. 36.5%; p = 0.46). Conversely, non-top-quality embryos achieved a 52% higher live birth rate (36.8% vs. 24.1%; p = 0.017) through CB-SETs as compared to FC-SETs. In conclusion, embryo quality must be controlled when alternative ET strategies are compared. FC-SETs can benefit patients and laboratory logistics when a top-quality embryo is produced, whereas a freeze-all strategy with CB-SETs may favor patients producing only sub-optimal embryos.

## 1 Introduction

Embryo selection and transfer (ET) strategies greatly impact ICSI/IVF results, as well as laboratory logistics and treatment cost. Although embryo selection still predominantly relies on static morphological evaluation, non-invasive approaches assessing embryo developmental competence, such as time-lapse monitoring (TLM), metabolic imaging and non-invasive PGT-A (pre-implantation genetic testing for aneuploidy), have been substantially improved and progressively incorporated in IVF/ICSI practice (1–3).

Time-lapse monitoring has emerged around twenty-five years ago as a promising tool to improve embryo selection accuracy. However, despite its increasing utilization its potential to improve live birth rates in ICSI/IVF practice has not yet been convincingly demonstrated (1, 4). Recently, by comparing ICSI/IVF outcomes of predominantly young patients (34.3 years old on average) subjected to transfers after either classical embryo morphological selection or embryo ranking with a generic TLM-based protocol, a robust RCT called into question the widespread application of TLM-assisted embryo selection (5). These findings/conclusions are not surprising since previous studies had already indicated that embryo morphokinetics varies with patient profile and culture conditions, making inhouse algorithm development or validation essential to achieve optimal accuracy in TLM-assisted embryo selection (6–9). In our practice, early morphokinetics has shown to be more accurate than embryo morphology to predict live birth achievement following cleavage stage transfers (10), which motivated us to develop an inhouse morphokinetic algorithm (KINEVO) for early embryo selection. The first objective of this study was to assess the efficacy of this algorithm in distinguishing embryos with higher competence to achieve a live birth following fresh cleavage stage transfers.

One of the key choices in the definition of ET strategy concerns embryo developmental stage. Over the last two decades, as culture systems evolved, ET has progressively moved from cleavage to blastocyst stage (11–13). Previous studies suggest that fresh blastocyst-stage transfers can provide higher live birth rates as compared to fresh cleavage-stage transfers, although the impact of extended embryo culture on cumulative live births, particularly in the face of low embryo availability, remains to be clarified. The apparent superior performance of blastocyst transfers has been interpreted as a consequence of deselection of poor-quality embryos during extended culture and better endometrial-embryonic synchrony (12, 14).

Another key decision in the definition of ET strategy is the choice between an immediate/fresh transfer or a post-cryopreservation transfer (freeze-all strategy), the last avoiding the negative impact of ovarian stimulation on endometrial receptivity and favoring the control of OHSS (ovarian hyperstimulation syndrome) risk. Collectively, the literature suggests that, although the freeze-all strategy appears not to significantly change the outcomes of normal responders and to negatively impact the cumulative live birth rates of poor-prognosis patients, it can benefit hyper-responders and good-prognosis patients (15–24).

By combining the observations above, one can thus conclude that good-prognosis patients should achieve optimal live birth rates through post-cryopreservation blastocyst transfers. Notably, however, even though it is well known that embryo quality impacts implantation and live birth achievement (8, 10, 25, 26), its potential influence on the comparative efficacy of alternative ET strategies has not been addressed or adequately controlled for in previous studies. Therefore, after demonstrating the efficacy of our Kinetic Evolution algorithm (KINEVO) in identifying cleavage-stage embryos with higher competence to achieve a live birth, we used this algorithm as an embryo ranking system to accomplish the second and principal objective of this study, i.e., to test the hypothesis that top-morphokinetic quality embryos from good prognosis patients can achieve optimal live birth rates if transferred fresh at the cleavage stage, thus not benefitting from extended culture combined with blastocyst cryopreservation and cycle segmentation. Conversely, we hypothesized that embryos with non-top morphokinetic quality would achieve higher live birth rates if cultured until the blastocyst stage, cryopreserved and transferred in a subsequent cycle.

## 2 Materials and methods

### 2.1 Local, patients and experimental design

This retrospective study was conducted at the Biogenesi Reproductive Medicine Centre, Monza, Italy, from 2018 to 2023. Approval from the local ethical committee for observational/non-interventionist studies assessing the impact of cycle and treatment characteristics on oocyte quality/recovery and IVF outcomes was previously obtained. All patients gave informed written consent, and the data were anonymized before analysis to protect participants’ identity.

To assess the efficacy of our KINEVO algorithm (**Supplementary Figure 1**) in distinguishing cleavage stage embryos competent to achieve a live birth following day 2/3 fresh transfers, 565 embryos, each of them provided by a single ICSI/IVF patient achieving a fresh cleavage stage single transfer (FC-SET), were classified according to their morphokinetic quality (KINEVO scores: A+, A-, B+, B-, C+, C-, D+, D-). In the present study, all fresh transfers were performed at cleavage stage (Day 2/3), whereas all cryopreserved transfers were 1^st^ attempt of frozen blastocyst (Day 5) derived from patients underwent a “freeze all approach” for different reasons. No fresh blastocyst transfers or cryopreserved cleavage-stage embryo transfers were included. Patient profiles and clinical outcomes were compared between FC-SETs (days 2/3) of A (top quality; A+/A-; n=345) and non-A embryos (non-top quality; n=220; **Table 1**). To control for the interference of potential confounders in the analysis, the association between KINEVO score (A *vs.* non-A) and live birth was assessed by a multivariate analysis including couple characteristics possibly affecting live birth as covariates (**Table 2**).

**Table 1:** Parental characteristics, morphokinetic stratification and treatment outcomes of embryos grouped according to morphokinetic quality score and subjected to fresh cleavage stage transfers (FC-SETs).

Score groups	Overall	A+	A-	B	C	D	A	Non-A	p (A vs. non-A)
N. of embryos	565 (100%)	291 (51.5%)	54 (9.6%)	22 (3.9%)	6 (1.1%)	192 (33.9%)	345 (61.1%)	220 (38.9%)	
Parental characteristics:									
Mat. age (years)	34.7 ± 4.1	34.2 ± 4.1	35.1 ± 4.0	35.0 ± 3.9	36.1 ± 3.9	35.4 ± 4.2	34.3 ± 4.1	35.4 ± 4.1	0.008
Pat. age (years)	38.1 ± 5.6	37.7 ± 5.4	37.6 ± 5.3	38.7 ± 5.7	38.0 ± 5.4	38.8 ± 5.8	37.7 ± 5.4	38.8 ± 5.8	0.040
AMH (ng/mL)	2.7 ± 1.0	2.7 ± 1.0	3.0 ± 1.0	2.5 ± 0.8	3.1 ± 1.2	2.7 ± 1.0	2.7 ± 1.0	2.7 ± 1.0	0.655
Basal FSH (IU/L)	7.4 ± 2.2	7.4 ± 2.1	7.7 ± 2.4	6.8 ± 1.8	6.9 ± 1.6	7.6 ± 2.4	7.4 ± 2.2	7.5 ± 2.3	0.405
Mat. BMI (kg/m²)	22.6 ± 2.5	22.6 ± 3.5	23.1 ± 3.4	23.2 ± 4.7	24.7 ± 2.4	22.2 ± 3.5	22.7 ± 3.4	22.4 ± 3.7	0.159
Morphokinetic score distribution:									
A+	51.5% (291)	100% (291)					84.3% (291)		NA
A-	9.6% (54)		100% (54)				15.7% (54)		
B+	1.0% (6)			27.3% (6)				2.7% (6)	
B-	2.8% (16)			72.7% (16)				7.3% (16)	
C+	0.9% (5)				83.3% (5)			2.3% (5)	
C-	0.2% (1)				16.7% (1)			0.5% (1)	
D+	2.8% (16)					8.3% (16)		7.3% (16)	
D-	31.2% (176)					91.7% (176)		80.0% (176)	
Treatment outcomes:									
Clinical Pregnancy	35.6% (201/565)	41.9% (122/291)	38.9% (21/54)	27.3% (6/22)	0.0% (0/6)	27.1% (52/192)	41.5% (143/345)	26.4% (58/220)	<0.0001
Miscarriage	9.5% (19/201)	11.5% (14/122)	4.8% (1/21)	16.7% (1/6)	0.0% (0/0)	5.8% (3/52)	10.5% (15/143)	6.9% (4/58)	0.309
Live Birth	31.7% (179/565)	36.4% (106/291)	37.0% (20/54)	22.7% (5/22)	0% (0/6)	25.0% (48/192)	36.5% (126/345)	24.1% (53/220)	0.001

Clinical pregnancy and live birth outcomes refer to rates per embryo transfer. NA, not applicable.

**Table 2:** Association of morphokinetic score (A vs. non-A) and potentially confounding variables (maternal age, paternal age, maternal AMH and maternal BMI) with live birth achievement following fresh cleavage stage transfers (FC-SETs).

Variable	Univariate OR (95% CI)	Univariate p	Multivariate OR (95% CI)	Multivariate p
Morphokinetic score (A vs. non-A)	1.81 (1.24–2.64)	0.002	1.62 (1.09–2.40)	0.015
Maternal Age	0.86 (0.82–0.90)	<0.0001	0.88 (0.83–0.93)	<0.0001
Paternal Age	0.93 (0.89–0.96)	<0.0001	0.99 (0.94–1.03)	0.494
Maternal AMH	1.07 (0.89–1.28)	0.46		
Maternal BMI	0.97 (0.92–1.02)	0.273		

To test the hypothesis that embryos with top morphokinetic quality can achieve equivalent outcomes through either a FC-SET or a cryopreserved blastocyst single transfer (CB-SET), clinical pregnancy, miscarriage and live birth rates provided by 610 KINEVO score A embryos transferred with either of these strategies (n=345 FC-SETs; n=265 CB-SETs) were compared (**Table 3**). In parallel, to test the hypothesis that embryos with inferior morphokinetic quality would achieve superior outcomes if subjected to extended culture followed by a CB-SET, clinical pregnancy, abortion and live birth rates from 315 non-A embryos (B+/B-/C+/C-/D+/D-) grouped according to ET strategy were compared (n=220 FC-SETs, n=95 CB-SETs; **Table 3**).

**Table 3:** Effect of embryo transfer strategy [FC-SET (fresh cleavage stage single transfer) vs. CB-SET (cryopreserved blastocyst single transfer)] on ICSI/IVF outcomes obtained with embryos grouped according to morphokinetic quality [Overall, A (top quality) and non-A morphokinetic scores].

Groups	FC-SET Overall§	CB-SET Overall§	FC-SET A*	CB-SET A*	FC-SET Non-A†	CB-SET Non-A†	p§	p*	p†
N. of embryos	565	360	345	265	220	95			
Parental characteristics:									
Mat. age (years)	34.7 ± 4.1	35.2 ± 4.2	34.3 ± 4.1	35.1 ± 4.2	35.3 ± 4.1	35.5 ± 4.0	0.082	0.026	0.771
Pat. age (years)	38.1 ± 5.6	38.3 ± 5.1	37.7 ± 5.4	38.2 ± 5.1	38.8 ± 5.8	38.5 ± 5.1	0.459	0.142	0.648
AMH (ng/mL)	2.7 ± 1.0	3.3 ± 1.0	2.7 ± 1.0	3.3 ± 1.0	2.7 ± 1.0	3.2 ± 1.0	<0.0001	<0.0001	<0.0001
Basal FSH (IU/L)	7.4 ± 2.2	7.0 ± 1.9	7.4 ± 2.2	7.0 ± 2.0	7.5 ± 2.3	7.0 ± 1.8	0.0008	0.012	0.032
Mat. BMI (kg/m²)	22.6 ± 2.5	22.2 ± 3.2	22.7 ± 3.5	22.0 ± 3.2	22.4 ± 3.7	22.6 ± 3.2	0.109	0.011	0.349
Morphokinetic classification:									
A+	51.5% (291)	67.2% (242)	84.3% (291)	91.3% (242)			<0.0001	0.006	0.028
A-	9.6% (54)	6.4% (23)	15.7% (54)	8.7% (23)					
B+	1.0% (6)	1.9% (7)			2.7% (6)	7.4% (7)			
B-	2.8% (16)	3.9% (14)			7.3% (16)	14.7% (14)			
C+	0.9% (5)	1.4% (5)			2.3% (5)	5.3% (5)			
C-	0.2% (1)	0.3% (1)			0.4% (1)	1.0% (1)			
D+	2.8% (16)	1.4% (5)			7.3% (16)	5.3% (5)			
D-	31.2% (176)	17.5% (63)			80.0% (176)	66.3% (63)			
Treatment outcomes:									
Clin. Pregnancy	35.6% (201/565)	47.5% (171/360)	41.4% (143/345)	47.5% (126/265)	26.4% (58/220)	47.4% (45/95)	<0.0001	0.082	<0.0001
Miscarriage	9.5% (19/201)	21.6% (37/171)	10.5% (15/143)	21.4% (27/126)	6.9% (4/58)	22.2% (10/45)	0.001	0.011	0.025
Live Birth	31.7% (179/565)	37.2% (134/360)	36.5% (126/345)	37.4% (99/265)	24.1% (53/220)	36.8% (35/95)	0.052	0.46	0.017

Clinical pregnancy and live birth outcomes refer to rates per embryo transfer.

In order to homogenize maternal profiles across experimental groups/transfer strategies, only patients with serum AMH concentrations between 1.5 and 5 ng/mL were included in the study. The interference of potential confounders in the associations between transfer strategy and clinical outcomes was controlled for by a multivariate analysis including maternal and paternal characteristics possibly affecting the outcomes as covariates (detailed in “Statistical Analysis”). In accordance with our local practice, only embryos from good prognosis patients under risk of ovarian hyperstimulation syndrome (OHSS) or presenting progesterone serum concentrations above 1.5 ng/mL on the day of trigger were subjected to CB-SETs.

The primary outcome of the present study was live birth rate per single elective transfer (number of live births divided by the number of embryo transfers), while secondary outcomes were clinical pregnancy rate per transfer (number of fetuses presenting heartbeat at 7 weeks of gestation divided by the number of transfers) and miscarriage rate (number of pregnancy losses from clinical pregnancy diagnosis to term divided by the number of clinical pregnancies).

### 2.2 Morphokinetic evaluation and ranking

All embryos were cultured in an integrated embryo culture TLM system (EmbryoScope™ Time-lapse System; Vitrolife, Göteborg, Sweden) and the EmbryoViewer image analysis software (Vitrolife, Göteborg, Sweden) was used to determine in hours post-insemination the following morphokinetic parameters: tPNf, time of pronuclear fading; t2, time at which two separate and distinct cells were identified; t3, time at which a three-blastomeres embryo was identified; t4, time at which a four-blastomeres embryo was identified.

Embryos were classified in eight KINEVO scores (A+; A-; B+; B-; C+; C-; D+; D-) using an algorithm developed from previous observations of our Fertility Centre indicating that morphokinetics is more accurate than morphology to drive early embryo selection (10). The algorithm consists in a chronological classification sequence using the median of the three morphokinetic parameters (tPNF, t2 and t4) found to be more significantly associated with live birth as a cut-off to distinguish faster from slower embryos, culminating in the eight aforementioned morphokinetic scores (**Supplementary Figure 1**). Embryos with t2 = t3 were classified as Uniparental Direct Cleavage (DUC1) and were excluded from the analysis.

### 2.3 Ovarian stimulation and embryo production/transfer

**Figure 1:**
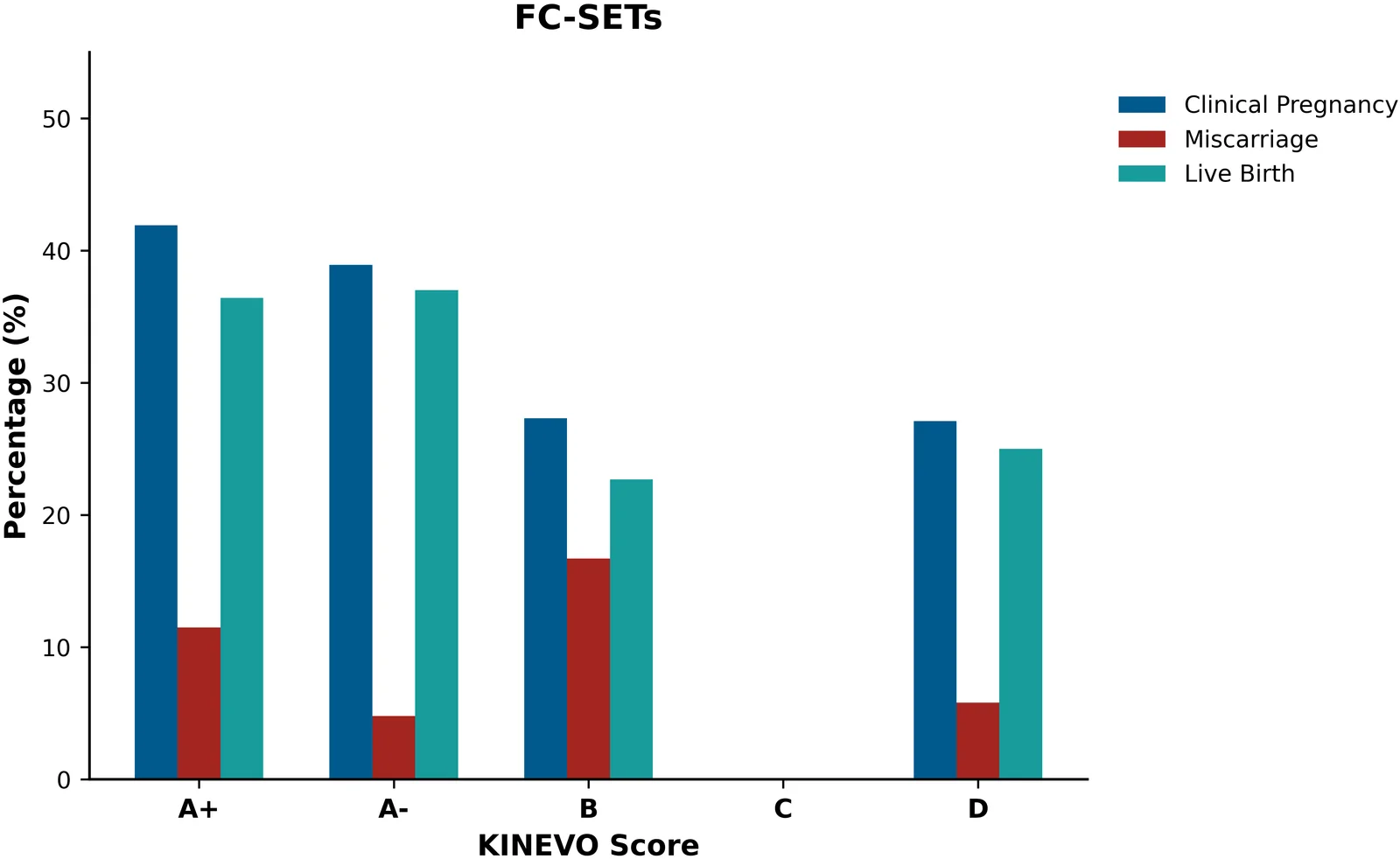
Treatment outcomes of embryos grouped according to morphokinetic quality score and subjected to fresh cleavage stage transfers (FC-SETs).

**Figure 2:**
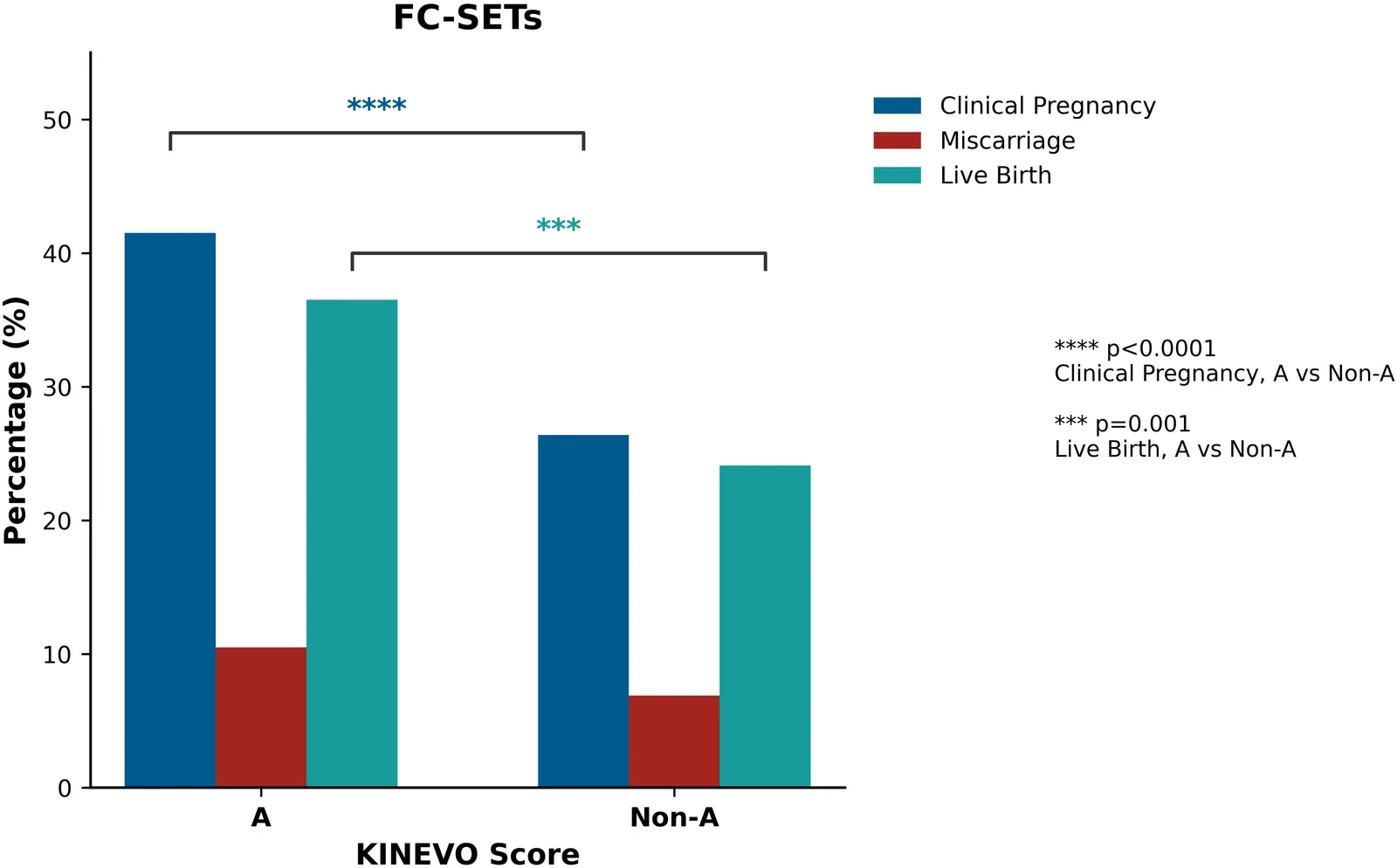
Treatment outcomes of A score vs Non-A score embryos subjected to fresh cleavage stage transfers (FC-SETs). **** p < 0.0001 indicates clinical pregnancy rate, A versus non-A; *** p = 0.001 indicates live birth rate, A versus non-A.

Ovarian stimulation was induced with rFSH or different associations of FSH, HMG, and LH in flexible antagonist or short agonist protocols (**Figures 1**, **2**). Hormone dose was defined and adjusted during stimulation according to patient profile and response. Oocyte maturation was triggered with either hCG or with a GnRH agonist 36 h prior to oocyte pickup, when at least three follicles ≥ 17–18 mm in diameter were first observed by ultrasound monitoring.

ICSI/IVF and embryo culture were performed according to the local routine as previously described (7); the same embryo culture medium was used throughout the study. Only single transfers of cleavage stage fresh embryos (FC-SET) or cryopreserved/thawed blastocysts (CB-SET) were included in the study. CB-SETs were preceded by endometrial preparation starting with estradiol administration (6-8mg/day) from cycle day 2 or 3. On the day endometrial thickness reached ≥7mm, progesterone administration was started (800mg intravaginally or 600mg intravaginally + 25mg subcutaneously daily), and ET was performed six days later. Clinical pregnancy was diagnosed 7 weeks later by ultrasound monitoring.

### 2.4 Statistical analysis

Throughout the study, continuous variables are presented as means and standard deviations, while categorical variables are described as percentages. Differences between continuous variables were assessed with the Wilcoxon sum rank test, whereas categorial variables were compared with the Fisher’s exact test.

The interference of potential confounders in the associations between KINEVO score (A *vs.* non-A) and live birth, and between transfer strategy (FC-SET *vs*. CB-SET) and clinical outcomes (live birth and miscarriage occurrence) was controlled for by univariate/multivariate logistic analyses including maternal and paternal characteristics possibly impacting the dependent variable as covariates (maternal and paternal age, maternal AMH and BMI, endometriosis and male infertility factor). Only covariates significantly (*p* < 0.05) or tending to be significantly (*p*≥0.05 and ≤0.1) associated with the dependent variable in the univariate analysis were included in the multivariate analysis.

The statistical analysis was performed using the Stata Software 9.0 (Stata Corporation, College Station, Texas, USA). Differences with *p* < 0.05 were considered significant, while those with p between 0.05 and 0.1 were considered statistical tendencies.

## 3 Results

The distribution of the 565 embryos utilized to assess the algorithm’s efficacy in different KINEVO score groups, together with major parental characteristics and treatment outcomes, is shown in **Table 1**. The majority of the embryos was classified as either A (top quality; n=345, 61.1%) or D (lowest quality; n=192, 33.9%), while a markedly lower percentage presented intermediate quality scores (B and C; n=28, 4.9%). Top quality embryos (score A) were provided by younger mothers (*p* = 0.008) and fathers (*p* = 0.04) as compared to non-top quality (non-A scores) embryos but did not differ regarding maternal AMH and BMI. Pregnancy and live birth rates provided by FC-SETs of score A embryos were 57% (*p* < 0.0001) and 51% (*p* = 0.001) higher than those provided by FC-SETs of non-A embryos, respectively. The multivariate analysis (**Table 2**) revealed that, in FC-SETs, the only variables independently associated with live birth were maternal age (OR = 0.88, 95% CI: 0.83-0.93, *p* < 0.0001) and KINEVO score (A *vs.* non-A; OR = 1.62, 95% CI: 1.09-2.40, *p* = 0.015).

The distribution of the 925 embryos utilized to assess the impact of ET strategy (FC-SET *vs.* CB-SET) on live birth achievement in different KINEVO quality groups (overall, A and non-A embryos), accompanied by major parental characteristics and treatment outcomes, is presented in **Table 3**. A higher proportion of top-quality embryos was transferred in CB-SETs as compared to FC-SETs; a score A embryo was transferred in 61.1% of the FC-SETs and in 73.6% of the CB-SETs (*p* < 0.0001). In all KINEVO quality groups (“overall”, “top quality/A score” and “non-top quality/non-A score”), CB-SETs were associated with higher AMH (*p* < 0.0001) and lower basal FSH (*p* < 0.035) maternal serum concentrations. In transfers of top-quality embryos (score A), but not in transfers of non-top-quality embryos (non-A), maternal age was significantly higher in CB-SETs (35.1 ± 4.2 years) as compared to FC-SETs (34.3 ± 4.1 years; *p* = 0.026).

In overall transfers, regardless of embryo quality, CB-SETs achieved a higher clinical pregnancy rate as compared to FC-SETs (47.5% *vs*. 35.6%; *p* < 0.0001), despite its association with a higher miscarriage rate (21.6% *vs*. 9.5%; *p* = 0.001); these effects were accompanied by a tendency of more frequent live birth occurrence following CB-SETs (37.2% *vs*. 31.7%; *p* = 0.052; **Table 3**).

Alternatively, in the sub-analysis including only transfers of top morphokinetic quality embryos, while clinical pregnancy achievement tended to be more frequent in CB-SET (47.5% *vs*. 41.4%; *p* = 0.082), miscarriage occurrence was significantly lower in FC-SET (21.4% *vs*. 10.5%; *p* = 0.011). Despite these evidences of different performances regarding implantation and pregnancy maintenance, live birth rates did not differ between the CB-SET and FC-SET strategies when a top-quality embryo was transferred (37.4% vs. 36.5%; *p* = 0.46; **Table 3**). Furthermore, the multivariate analysis confirmed the significant positive association between CB-SET and miscarriage (OR 2.09, 95%CI: 1.12-3.91, *p* = 0.021) even after correcting for potential confounders such as maternal age, paternal age, maternal AMH and BMI, endometriosis, and male subfertility (**Table 4**).

**Table 4:** Association of miscarriage occurrence with embryo transfer strategy [CB-SET (cryopreserved blastocyst single transfer) vs. FC-SET (fresh cleavage stage single transfer)] and potentially confounding variables (maternal age, paternal age, maternal AMH, maternal BMI, endometriosis and male infertility factor) in cycles transferring a top-morphokinetic quality embryo (score A).

Variable	Univariate OR (95% CI)	Univariate p	Multivariate OR (95% CI)	Multivariate p
Transfer strategy (CB-SET vs. FC-SET)	2.64 (1.46–4.80)	<0.0001	2.09 (1.12–3.91)	0.021
Maternal Age	1.22 (1.12–1.33)	<0.0001	1.23 (1.10–1.37)	<0.0001
Paternal Age	1.06 (1.00–1.12)	0.057	0.97 (0.90–1.05)	0.468
Maternal AMH	1.05 (0.79–1.39)	0.746		
Maternal BMI	0.92 (0.84–1.01)	0.076	0.94 (0.85–1.03)	0.177
Endometriosis	2.41 (0.89–6.50)	0.083	2.65 (0.93–7.61)	0.069
Male infertility factor	0.78 (0.43–1.44)	0.431		

**Figure 3:**
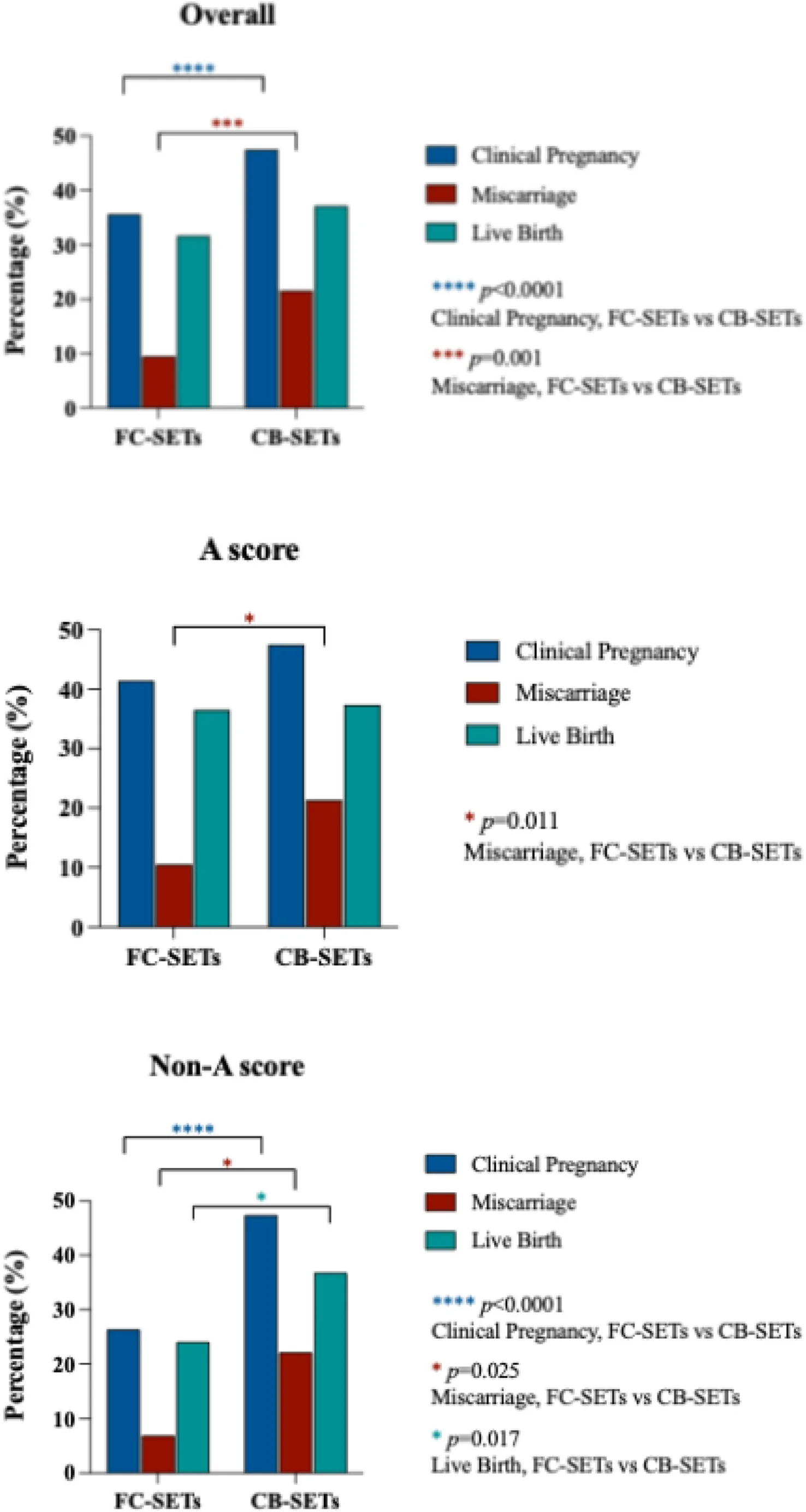
Comparison of treatment outcomes between FC-SETs (fresh cleavage-stage single transfers) and CB-SETs (cryopreserved blastocyst single transfers), with embryos grouped according to morphokinetic quality: overall, A-score (top-quality) and non-A-score embryos. **** p = 0.0001; *** p = 0.001; * p = 0.011, 0.025 or 0.017, as specified in the figure legend and annotation.

In the sub-analysis including only transfers of non-top-quality embryos, CB-SETs achieved an approximately 80% higher clinical pregnancy rate per transfer than that observed following FC-SETs (47.4% vs. 26.4%; *p* < 0.0001). Although miscarriage occurrence was once again higher following CB-SETs (22.2% vs. 6.9%; *p* = 0.025), live birth achievement per transfer was approximately 52% more frequent after CB-SET as compared to FC-SET of non-top-quality embryos (36.8% *vs.* 24.1%; *p* = 0.017; **Table 3**; **Figure 3**).

## 4 Discussion

The use of frozen blastocyst transfers, commonly referred to as the freeze-all strategy, has substantially increased in ICSI/IVF practice over the last decade (15, 16, 18). Taken together, previous studies suggest that good prognosis patients benefit from the freeze all strategy (15–24), although it is still not known whether the comparative efficacy of this strategy may vary according to the quality of the embryo(s) available for transfer. Herein, we provide novel evidence that embryo morphokinetic quality is a valuable parameter for the definition of the ET strategy in order to optimize IVF/ICSI live birth achievement.

The data depicting the performance of our KINEVO algorithm, the first objective of the present study, revealed that couples achieving a transfer of a top quality (score A) embryo present lower maternal age. Alternatively, our first multivariate analysis indicates that, in the present dataset, the apparent negative relationship between paternal age and availability of a top-quality embryo reflects, in great part, the association between paternal and maternal age. The influence of paternal age on embryo morphokinetics, however, cannot be ruled out. Embryo morphokinetics was reported to slow down as paternal age advances in a previous study assessing a more heterogeneous group of ICSI patients (27). The present findings are nevertheless in line with the well stablished concept that maternal age is a major determinant of gamete/embryo quality (28), as well as with previous evidence that maternal age is associated with slower embryo morphokinetics (8). Most importantly, our first multivariate analysis confirmed that the association of embryo morphokinetics with live birth achievement, and thus the performance of KINEVO is independent of parental age and other potential confounding variables (maternal BMI and AMH). The efficacy of KINEVO in identifying embryos with a higher chance to achieve a live birth following a FC-SET agrees with previous converging evidence that faster embryos do hold higher developmental competence (10, 26, 29, 30).

In the present study, CB-SETs promoted overall higher pregnancy rates, accompanied by a trend of higher live birth rates, as compared to FC-SETs. This was associated with higher occurrence of transfers of top-quality embryos in the CB-SET group, which we interpret as a consequence of higher maternal AMH serum levels, higher oocyte yields and thus higher availability of score A embryos in CB-SET patients (**Table 3**). One can thus conclude that the overall superior outcomes achieved with CB-SET reflect in great part the quality of the embryo transferred, and not necessarily the superior performance of the ET strategy itself. Importantly, these observations highlight the need to control the data, not only for the influence of patient variables, but also for the impact of embryo quality, when comparing the performance of alternative ET strategies. It is also important to point out that embryo morphology appears not to constitute a sufficiently accurate indicator of embryo quality for such control; we have previously reported that, in our practice, around 20% of the embryos with top morphology do not present top morphokinetics, the last characteristic being clearly more predictive of a live birth (10).

In agreement with the central hypothesis of the study, CB-SETs and FC-SETs provided equivalent live birth rates when a top-quality embryo was transferred. Interestingly, however, whilst clinical pregnancy was tendentially more frequent following CB-SETs, miscarriage incidence was significantly lower following FC-SETs of top-quality embryos. The trend towards higher pregnancy rates following CB-SETs possibly reflects, at least in part, a more favorable uterine environment for implantation in the absence of the impact of ovarian stimulation on the endometrium, as well as better endometrial-embryonic synchrony (12, 14, 31). Of note, however, CB-SETs resulted in a more than twice higher miscarriage incidence as compared to FC-SETs, regardless of the quality of the embryo transferred; consistent results are reported for overall, top-quality (score A) and inferior-quality (scores B, C and D) groups (**Table 3**). The reasons underlying this finding are not entirely clear and may combine both the impact of embryo cryopreservation and epigenetic disturbances induced by prolonged embryo culture. Nonetheless, while previous studies converge to suggest that embryo cryopreservation does not increase miscarriage incidence (17, 24, 32), higher miscarriage occurrence was associated with blastocyst as compared to cleavage stage transfers in the most recent and robust metanalysis available (12, 14). The association of prolonged embryo culture with increased miscarriage incidence is supported by previous basic/mechanistic data. Several studies using animal models have consistently indicated that culture conditions can epigenetically alter embryo DNA methylation and transcription patterns, compromising the expression of functionally crucial imprinted genes in the placenta (33–38).

As previously mentioned, our findings indicate that patients providing top quality embryos do not benefit from prolonged embryo culture combined with blastocyst cryopreservation and cycle segmentation; live birth achievement following transfers of score A embryos did not differ between FC-SET and CB-SET in the present study. Of note, however, a considerable percentage of top-quality embryos is lost during extended culture (15.4% in the present study). Therefore, apart from increasing time to pregnancy and patient anxiety, deciding for the CB-SET strategy may reduce the chances of reaching an ET and thus a live birth, particularly when few top-quality embryos are available on Day 2/3. Alternatively, the present data indicate that patients providing only non-top quality embryos may benefit from the CB-SET strategy, despite the losses during prolonged embryo culture (26.9% of the non-A embryos subjected to extended culture on day 2 did not reach the blastocyst stage in the present study). Importantly, even if the CB-SET strategy does not appear to drastically impact live birth rates per cleavage stage embryo produced, the significantly higher live birth rate per transfer obtained with CB-SET could justify its utilization to avoid the financial and psychological burdens associated with failed transfers, when only non-top quality embryos are available.

This study is limited by its retrospective nature and, even if the interference of major confounding variables was controlled for by multivariate analyses, we cannot rule out the influence of other possibly non-identified confounding factors. The fact that the groups under comparison (FC-SET *vs.* CB-SET) differ in more than one aspect (embryo stage at transfer and embryo cryopreservation/cycle segmentation) may also be considered a fragility of the study, since both factors can contribute for the differences reported. In particular, the influence of embryo quality on the performance of fresh blastocyst transfers and frozen cleavage stage transfers in comparison with FC-SET and CB-SET remains to be investigated. However, although these limitations inspire care in the interpretation of the present data, they do not invalidate the novel central hypothesis of the study, nor its results and conclusions/implications.

In conclusion, the present findings constitute reassuring evidence that early embryo morphokinetics reflects developmental competence. Most importantly, this study demonstrates for the first time that embryo quality largely impacts the comparative performance of different ET strategies; patients providing only non-top quality embryos, but not those providing top quality embryos, may benefit from extended culture to the blastocyst stage combined with cryopreservation and cycle segmentation. Alternatively, fresh cleavage stage transfers may constitute a valid strategy to decrease miscarriage occurrence without reducing live birth chances in good prognosis patients providing high quality embryos.

## Data Availability

The original contributions presented in the study are included in the article/**Supplementary Material**. Further inquiries can be directed to the corresponding authors.
